# *AKAP9*-Related Channelopathy: Novel Pathogenic Variant and Review of the Literature

**DOI:** 10.3390/genes13112167

**Published:** 2022-11-20

**Authors:** Minh-Tuan Huynh, Alexis Proust, Jérôme Bouligand, Elena Popescu

**Affiliations:** 1 Centre Hospitalier du Havre, Unité de Génétique Clinique, 29 Avenue Pierre Mendès-France, 76290 Montivilliers, France; 2 Laboratoire de Génétique Moléculaire, Pharmacogénétique et Hormonologie, Hôpital Bicêtre, APHP Université Paris Saclay, 94270 Le Kremlin-Bicêtre, France; Inserm UMR_S 1185, Faculté de Médecine Paris Saclay, Université Paris Saclay, 94270 Le Kremlin-Bicêtre, France; 3 Centre Hospitalier du Havre, Service de Cardiologie, 29 Avenue Pierre Mendès-France, 76290 Montivilliers, France

**Keywords:** *AKAP9* haploinsufficiency, long QT syndrome 11, sudden cardiac arrest, novel frameshift, variant

## Abstract

Disease-associated pathogenic variants in the A-Kinase Anchor Protein 9 (*AKAP9*) (MIM *604001) have been recently identified in patients with autosomal dominant long QT syndrome 11 (MIM #611820), lethal arrhythmia (ventricular fibrillation, polymorphic ventricular tachycardia), Brugada syndrome, and sudden unexpected death. However, *AKAP9* sequence variations were rarely reported and *AKAP9* was classified as a “disputed evidence” gene to support disease causation due to the insufficient genetic evidence and a limited number of reported *AKAP9*-mutated patients. Here, we describe a 47-year-old male carrying a novel frameshift *AKAP9* pathogenic variant who presented recurrent syncopal attacks and sudden cardiac arrest that required a semi-automatic external defibrillator implant and an electric shock treatment of ventricular arrhythmia. This study provides insight into the mechanism underlying cardiac arrest and confirms that *AKAP9* loss-of-function variants predispose to serious, life-threatening ventricular arrhythmias.

## 1. Introduction

Long QT syndrome (LQTS), an inherited arrhythmogenic disorder, is characterized by a prolonged QT-interval, QT interval lability, polymorphic ventricular tachycardia which may lead to recurrent syncopal episodes, cardiac arrest, and sudden cardiac death, mostly at a young age. Deleterious *AKAP9* pathogenic variants have been recently reported in patients with congenital LQTS type 11 (MIM #611820). To date, only five patients with LQTS harboring *AKAP9* pathogenic variants were documented [1,2,3,4]. However, some *AKAP9*-mutated patients also harbored a second genetic variant in channelopathy-associated genes [1]. In addition, publications of *AKAP9* were based on a candidate gene approach; there were no segregation of suspected pathogenic variants in multiple affected cases in large families, and case-control studies were performed leading to the absence of genetic evidence to support the causation of LQTS. Hence, the *AKAP9* gene was classified as a “disputed LQTS-causative gene” because of the insufficient genetic evidence to support disease causation [5]. We report here the case of a 47-year-old man with recurrent syncopal attack and cardiac arrest who harbored a novel *AKAP9* frameshift variant and in whom LQTS type 11 was diagnosed. Our study adds knowledge to the existing literature of LQTS type 11 and confirms that pathogenic *AKAP9* “loss-of-function” variants are associated with LQTS.

## 2. Materials and Methods

### 2.1. Targeted Next-Generation Sequencing

Genomic DNA was extracted from the whole blood of the family using standard methods (QIAGEN, Hilden city, Germany). We performed a targeted sequencing of 132 genes (supplemental material) associated with sudden unexpected death for the proband. Enrichment was performed with TWIST Technology to capture all coding regions and exon-intron junctions (±50 bps) followed by Illumina NextSeq500 (San Diego, CA, USA) 2 × 150 paired-end sequencings.

We obtained >99% of targeted regions covered more than 30 times and 99X mean depth. Raw data were analyzed using the SeqOne genomics interpretation platform. Variants were annotated according to the Human Genome Variation Society guidelines (HGVS), mapped to the Human Genome Build GRCh37/UCSC hg19, and classified according to the criteria of the American College of Medical Genetics and Genomics.

### 2.2. Sanger Sequencing

Specific primers were designed to amplify exon 31 of the *AKAP9* gene (NM_005751.5). PCR products were then sequenced using BigDye Terminator v.3.1 (Thermo Fisher Scientific, MA, USA).

### 2.3. MetaDome Analysis and Protein Structure Modeling

A graphic representation of the tolerance score of the affected Glu^2418^ of the *AKAP9* protein was obtained by the MetaDome web server (https://stuart.radboudumc.nl/metadome/ (accessed on 22 May 2019). Structural change modeling of the identified mutant Yotiao protein was performed using PyMOL packaged software (www.pymol.org/pymol (accessed on 22 May 2019).

## 3. Results

### 3.1. Clinical Report

A healthy 47-year-old man was admitted to the emergency department suffering from cardiac arrest. He had a personal medical history of recurrent syncopal attacks without a relevant family history of cardiac diseases.

The patient experienced his first syncope with self-limited loss of consciousness at work in October 2008 and was admitted to the Cardiology Department. Clinical examination showed weak peripheral pulses with the presence of breathing movements, hypertonia without spasticity, and loss of bladder control, followed by a brief episode of respiratory arrest with absent peripheral pulses. His fainting was quickly resolved with shallow breathing, tachycardia, and low blood pressure of 80/40 mmHg. All paraclinical examination results such as C-reactive protein (CRP), electroencephalogram (EEG), brain scanner, echocardiogram (ECG), cardiac ultrasound, and stress test were within normal limits. He also had a negative ajmaline challenge test. However, a tilt-table test demonstrated the presence of orthostatic hypotension with the introduction of a GUTRON treatment.

He had repeated episodes of fainting in November 2014 whilst working in the office and was again readmitted to the Cardiology Department. It was similar to his first hospital admission, and clinical examination was also unremarkable. ECG showed a sinus rhythm with a normal PR interval, a corrected QT interval (QTc) of 393 milliseconds, while 24 h Holter monitoring demonstrated a normal PR interval, a QTc interval of 450 to 487 milliseconds. An exercise stress test revealed a QTc calculated at rest of 418 milliseconds and a QTc of 442 milliseconds at 4 min. The diagnosis of vasovagal syncope was maintained and the patient continued treatment with GUTRON.

In November 2021, he fainted in the office with the presence of cardiac arrest and lost consciousness. A closed-chest cardiac massage was immediately performed in the absence of respiratory movements and unconsciousness (no blood flow was evaluated at 0 min, reaching a low blood flow at 7 min). In addition, a semi-automatic external defibrillator was also implemented, followed by electric shock, and repeated cardiac massage (Supplemental Figure S1).

**Figure 1:**
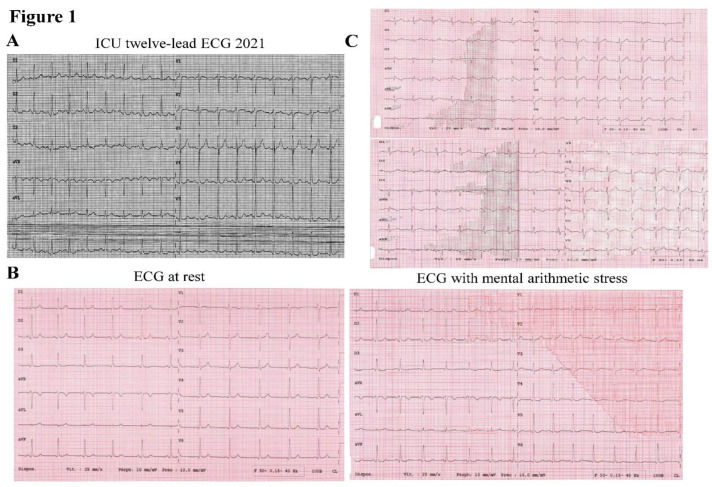
(**A**) ICU twelve-lead ECG showed a normal sinus rhythm, a normal PR interval at 160 milliseconds, a QRS complex, and a silent “prolonged” QTc interval of 436 milliseconds. (**B**) Repeated resting (left panel) and mental arithmetic stress ECG (right panel) showed QTc intervals of 400 and 457 milliseconds, respectively, which confirm the diagnosis of LQTS. (**C**) His father’s ECG at rest and during mental arithmetic stress showed a prolonged QTc of 460 (upper panel) and 485 milliseconds (lower panel).

He recovered consciousness but presented delirium and anterograde amnesia with stable hemodynamics. Coronary angiography showed no significant lesions, present peripheral pulses, a Glasgow score of 15, his twelve-lead ECG showed normal sinus rhythm, a PR interval of 160 milliseconds, a normal QRS complex, and a borderline QTc value of 436 milliseconds (Figure 1A). Moreover, repeated resting ECG and ECG during mental arithmetic stress showed a QTc value of 400 milliseconds and 457 milliseconds, respectively (Figure 1B). His father, a 73-year-old man, had no past medical history of cardiovascular disease. He complained of an episode of malaise in 2017 when driving his car. His twelve-lead ECG at rest and during mental arithmetic stress showed a prolonged QTc interval of 460 milliseconds and 485 milliseconds, respectively (Figure 1C).

### 3.2. Genetics Analysis Results

**Figure 2:**
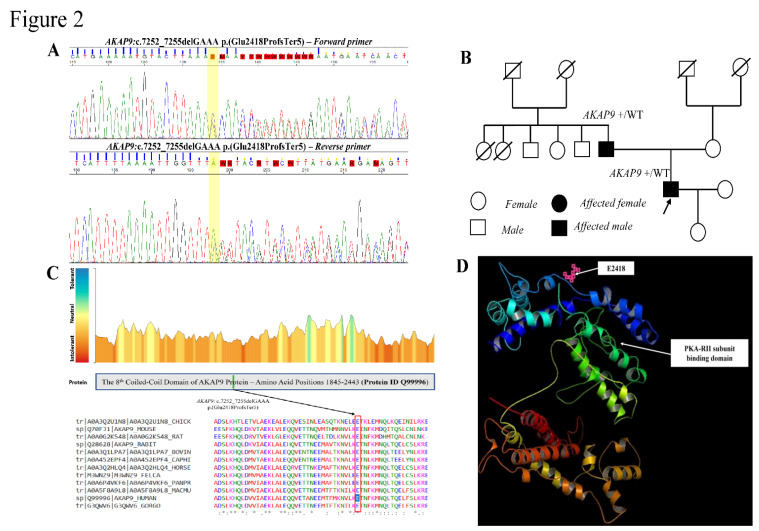
(**A**) Sanger sequencing showing a frameshift *AKAP9* variant c.7252_7255delGAAA p.(Glu2418Profs*5) in the propositus. (**B**) Familial segregation study showing the pathogenic *AKAP9* frameshift variant was inherited from an apparently normal father. (**C**) MetaDome showing a graphic representation of the tolerance scores of the affected Glu^2418^ highly conserved among orthologs. (**D**) PyMOL showing the Yotiao 3D protein structure, the frameshift variant *AKAP9*: c.7252_7255delGAAA p.(Glu2418Profs*5) leading to the production of truncated Yotiao protein lacking the PKA-RII subunit binding domain, resulting in reduced PKA-dependent phosphorylation of the Ser^27^ located in the N terminus of I_Ks_ α-subunit KCNQ1, which gives rise to the prolonged QTc interval.

Targeted next-generation sequencing of 132 genes associated with unexplained sudden death (Appendix A) revealed a heterozygous frameshift variant in exon 31 of the *AKAP9* gene (NM_005751.5): c.7252_7255delGAAA p.(Glu2418ProfsTer5) in the propositus. The frameshift *AKAP9* variant was confirmed by Sanger sequencing. A segregation study demonstrated that the pathogenic variant was of paternal origin (Figure 2A,B). The variant was absent in other healthy family members with normal ECG. Moreover, this variant has a low frequency of 1,067 × 10^−5^ (3/281134) and 0.000378% in the gnomAD and TOPMed bravo databases, respectively.

### 3.3. MetaDome Tolerance Score Analysis and Structural Yotiao Protein Modelling

The Glu^2418^ is also highly conserved during species and predicted to be intolerant to changes by MetaDome [6] (Figure. 2C). This frameshift variant introduces a premature codon stop potentially leading to the production of aberrant Yotiao transcripts degraded by nonsense-mediated RNA decay or synthesis of nonfunctional, truncated Yotiao protein lacking the critical PKA-RII binding domain (Figure 2D).

## 4. Discussion

The cardiac IKs channel, a major repolarization current, is composed of the α-subunit of the voltage-gated channel and a β-subunit encoded, respectively, by *KCNQ1* and *KCNE1,* whose activity is strictly controlled by the sympathetic nervous system. Pathogenic variants in the cardiac I_Ks_ channel α-subunit localized predominantly within the *KCNQ1*/*AKAP9* (Yotiao) binding domains were recently shown to cause Long QT Syndrome (LQTS) [3]. Yotiao is the smallest splicing variant of A-kinase Anchoring Protein 9, which forms a macromolecular complex with the voltage-gated K^+^ channel to regulate the functions of the cardiac I_Ks_ channel. Yotiao recruits Protein Kinase A via its PKA-RII binding domain to modulate the phosphorylation of the single Ser^27^ residue located in the KCNQ1 N terminus for tight control of I_Ks_ function.

Disease-associated pathogenic *AKAP9* variants were rarely reported and associated with a broad phenotypic spectrum, including LQTS type 11 [1,2,3,4], Brugada syndrome [7,8], unexplained sudden death [9,10,11,12], severe ventricular arrhythmia [13,14], and cardiomyopathy [15] (Table 1).

However, *AKAP9* is classified as a disputed LQTS-causative gene due to the absence of sufficient evidence to support disease causation [5]. Of interest, our report could confirm that pathogenic *AKAP9* variants were associated with lethal arrhythmias and cardiac sudden death. The proband displayed recurrent syncopal episodes and aborted cardiac arrest without a typically prolonged QTc interval. The challenges arise when *AKAP9*-mutated patients presenting with a QTc in the normal or borderline zone might be underdiagnosed if based solely on routine ECG. The patient’s 12-lead ECG in 2014 showed normal QTc values of 393 milliseconds, while 24 h Holter ECG revealed a “borderline” corrected QT interval prolongation of 442 milliseconds, making it difficult to reach a clinical diagnosis. The second twelve-lead ECG with a borderline QTc interval of 436 milliseconds in 2021 is also insufficient to warrant the diagnosis of LQTS. Indeed, 25% to 35% of “silent carriers” with a QTc < 440 milliseconds harbor a pathogenic variant in one of the known LQTS genes [16]. His father also carried the frameshift variant, but exhibited minor clinical features, indicating the clinical variability and incomplete penetrance of LQTS type 11.

On the other hand, it is worth noting that some *AKAP9*-mutated patients could also harbor other genetic alterations in the channelopathy-associated genes, suggesting complex multifactorial and polygenic traits [9]. Of note, the triggering elements such as the patient’s genetic background, environmental factors, gender, and genetic modifiers could contribute to inducing severe life-threatening ventricular arrhythmia. In fact, the patient is a financial auditor; his syncopal attacks occurred spontaneously under psychological stress when he tried to finish the company’s annual financial reports at the end of the year. Therefore, in this case, the patient also had obvious stressors prior to the recurrent syncope.

In conclusion, our study adds to the existing knowledge by reporting a novel pathogenic *AKAP9* “loss of function” variant associated with clinically “concealed” LQTS manifested by recurrent syncopal attacks and cardiac arrest. We also propose that molecular analysis of arrhythmia-related genes could be offered in patients with recurrent syncopal events, although there was an absence of a family history of sudden unexpected death. Taken together, these results reinforce that *AKAP9* haploinsufficiency predisposes to LQTS and other cardiac phenotypes with incomplete penetrance and variable clinical phenotypes.

## Figures and Tables

**Table 1:** Previously reported *AKAP9* variants associated with different cardiac phenotypes.

*AKAP9*	Nucleotide Change (c.DNA Nomenclature)	Protein Change	Exonic Localisation	Variant Type	Parent of Origin	Clinical Symptoms	Associated Disease(s)	Reference(s)
1	c.139C > T	p.(His47Tyr)	Exon 02	Missense	De novo	Syncope at 10-years-old, ECG: QTc >450 ms, and QTc max of 475 ms	LQTS *	[1]
2	c.2239G > A	p.(Glu747Lys)	Exon 08	Missense	N/A	Cyanotic syncope, sinus tachycardia of 110 bpm, and ECG: J-point elevations of 1–2 mm in V1-V3, with saddle-back morphology most prominent in V1 and V2.	Brugada syndrome	[7]
3	c.3673G > A	p.(Leu1150Phe)	Exon 09	Missense	N/A	QTc >570–580 ms, episodes of torsade de pointes (8 sec longest duration), and dual-chamber ICD implantation. Echocardiography: left ventricular hypertrophy	LQTS	[2]
4	c.3827G > A	p.(Arg1276Gln)	Exon 12	Missense	Maternal transmission	Unexplained sudden death (daughter)	USD *	[9]
5	c.3827G > A	p.(Arg1276Gln)	Exon 12	Missense	De novo	Unexplained sudden death (Mother)	USD *	[9]
6	c.3827G > A	p.(Arg1276Gln)	Exon 12	Missense	Maternal transmission	Positive flecainide, ECG, and EPS	Brugada syndrome	[8]
	c.8573A > G	p.(Tyr2858Cys)	Exon 33	Missense	De novo			
7	c.4826G > A	p.(Arg1609Lys)	Exon 18	Missense	Paternal transmission	Syncope at 7-years-old, QTc > 450 ms, and QTc max of 489 ms. ECG of proband’s father QTc > 450 ms	LQTS *	[1]
8	c.4826G > A	p.(Arg1609Lys)	Exon 18	Missense	Maternal transmission	Unexplained sudden death and family history of sudden death	USD *	[10]
9	c.4709C > T	p.(Ser1570Leu)	Exon 18	Missense	N/A	Syncope, QTc > 485 ms, and positive family history	LQTS	[3]
10	c.4927A > C	p.(Ile1643Leu)	Exon 19	Missense	Maternal transmission	Unexplained sudden death	USD *	[11]
11	c.6065A > G	p.(Gln2022Arg)	Exon 25	Missense	N/A	Seizures, QTc 485 ms, rising T waves, T-wave inversion in V1-V3, and notched wave in V4-V6	LQTS	[4]
12	c.6134A > G	p.(Asn2045Ser)	Exon 26	Missense	N/A	Sudden unexplained death and epilepsy	USD	[12]
13	c.7725A > C	p.(Gln2575His)	Exon 31	Missense	Paternal transmission	Unconsciousness, QTc 440 ms and QTc 560 ms, and fever	Episodes of torsade de pointes *	[13]
14	c.7438C > T	p.(Gln2480 *)	Exon 31	Nonsense	N/A	Episodes of recurrent ventricular fibrillation in the context of early repolarization syndrome and ICD implantation	Ventricular polymorphic tachycardia at rest	[14]
15	c.8656A > G	p.(Ile2886Val)	Exon 34	Missense	Paternal transmission	Positive flecainide and EPS, negative ECG, and family history of unexplained sudden death	Brugada syndrome	[8]
16	c.10303C > T	p.(Arg3435 *)	Exon 41	Nonsense	N/A	Palpitations, syncope on effort, 2D Echo: Right ventricule dilatation, biventricular enlargement at ventriculography, recurrent episodes of sustained ventricular tachycardia, and ventricular fibrillation discontinued by ICD shocks.	DCM	[15]
17	c.11610C > G	p.(Tyr3870 *)	Exon 49	Nonsense	N/A	Chest pain, non-obstructive hypertrophic cardiomyopathy (IVS thickness 22 mm), and ICD implantation	HCM	[15]

**LQTS:** Long QT Syndrome; **USD:** Unexplained Sudden Death; **DCM:** Dilated Cardiomyopathy; **HCM:** Hypertrophic Cardiomyopathy; **ICD:** Implantable cardioverter-defibrillator; * Presence of other variants in channelopathy-associated genes; **N/A:** Not Available.

## Data Availability

The data were submitted in LOVD databases: Individual #00416441 https://databases.lovd.nl/shared/individuals/00416441 (accessed on 22 May 2019).

## Supplementary Materials

The following are available online at https://www.mdpi.com/article/10.3390/genes13112167/s1, Figure S1: Showing the implantable cardioverter defibrillator shock record.

## Appendix A

The Gene Panel Includes 132 Genes Associated with the Unexplained Sudden Death and Congenital Long QT Syndrome.

*ABCC9 ACTC1 ACTN2 AKAP9 ALMS1 ALKP3 ANK2 ANKRD1 BAG3 BRAF CACNA1C CACNA2D1 CACNB2 CALM1 CALM2 CALM3 CALR3 CASQ2 CAV3 CAVIN4 CHRM2 CRYAB CSRP3 CTF1 CTNNA3 DES DMD DOLK DSC2 DSG2 DSP DTNA EMD EYA4 FBN2 FHL1 FKRP FKTN FLNC GAA GATA4 GATA5 GATA6 GATAD1 GJA5 GLA GNB5 GPD1L HCN4 HFE HRAS ILK JPH2 JUP KCNA5 KCND3 KCNE1 KCNE2 KCNE3 KCNE5 KCNH2 KCNJ2 KCNJ5 KCNJ8 KCNQ1 KRAS LAMA4 LAMP2 LDB3 LMNA LRRC10 LRP6 MAP2K1 MAP2K2 MIB1 MYBPC3 MYH6 MYH7 MYL2 MYL3 MYL4 MYLK2 MYOZ2 MYPN NEBL NEXN NKX2-5 NRAS PDLIM3 PKP2 PLN PPA2 PRDM16 PRKAG2 PSEN1 PSEN2 PTPN11 RAF1 RANGRF RMB20 RIT1 RYR2 SCN10A SCN1B SCN2B SCN3B SCN4B SCN5A SGCD SHOC2 SLC25A4 SNTA1 SOS1 TAZ TBX20 TCAP TECRL TGFB3 TGFBR2 TMEM43 TMPO TNNC1 TNNI3 TNNT2 TOR1AIP1 TPM1 TRDN TRPM4 TTN TTR TXNRD2 VCL*.

## References

[1] Maltese E.P., Orlova N., Krasikova E., Emelyanchik E., Cheremisina A., Kuscaeva A., Salmina A., Miotto R., Bonizzato A., Guerri G. (2017). Gene-targeted Analysis of Clinically Diagnosed Long QT Russian Families. Int. Heart. J..

[2] Bottigliero D., Monaco I., Santacroce R., Casavecchia G., Correale M., Guastafierro F., Leccese A., Cordisco G., Leva R., Trunzo R. (2019). Novel AKAP9 mutation and long QT syndrome in a patient with torsades des pointes. J. Interv. Card. Electrophysiol..

[3] Chen L., Marquardt L.M., Tester D.J., Sampson K.J., Ackerman M.J., Kass R.S. (2007). Mutation of an A-kinase anchoring protein causes long-QT syndrome. Proc. Natl. Acad. Sci. USA.

[4] Tse G., Lee S., Zhou J., Liu T., Wong C.K.I., Mak C., Mok N.S., Jeevaratnam K., Zhang Q., Cheng S.H. (2021). Territory-wide Chinese Cohort of Long QT syndrome: Random survival Forest and Cox Analyses. Front. Cardiovasc. Med..

[5] Arnon A., Novelli V., Amin S.A., Abiusi E., Care M., Nannenberg A.E., Feilotter H., Amenta S., Mazza D., Bikker H. (2020). An international, Multicentered, Evidence-Based Reappraisal of Genes Reported to Cause Congenital long QT Syndrome. Circulation.

[6] Wiel L., Baakman C., Gilissen D., Veltman J.A., Vriend G., Gilissen C. (2009). MetaDome: Pathogenicity analysis of genetic variants through aggregation of homologous human protein domains. Hum. Mut..

[7] Garris R., Vasudev R., Gupta P., Tiyyagura S., Shamoon F., Bikkina M. (2019). Brugada syndrome and AKAP9: Reconciling clinical findings with diagnostic uncertainty. J. Electrocardiol..

[8] Allegue C., Coll M., Mates J., Campuzano O., Iglesias A., Sobrino B., Brion M., Amigo J., Carracedo A., Brugada P. (2015). Genetic Analysis of Arrythmogenic Disease in the Era of NGS: The complexity of Clinical Decision-Making in Brugada Syndrome. PloS One.

[9] Li J.L., Wang B.Y., Qu F.P., Ma L., Liu K., Yang L., Nie J.S., Xi M.Y., Jia L.P., Tang X. (2020). Genetic analysis of Yunnan sudden unexplained death by whole exome sequencing in Southwest of China. J. Forensic. Leg. Med..

[10] Jaouadi H., Bouyacoub Y., Chabrak S., Kraoua L., Zaroui A., Elouej S., Nagara M., Dallali H., Delague V., Levy N. (2021). Multiallelic rare variants support an oligogenic origin of sudden cardiac death in the young. Herz.

[11] Campuzano O., Allegue C., Sarquella-Brugada G., Coll M., Mates J., Alcalde M., Ferrer-Costa C., Iglesias A., Brugada J., Brugada R. (2014). The role of clinical, genetic and segregation evaluation in sudden infant death. Forensic. Sci. Int..

[12] Neubauer J., Lecca R.M., Russo G., Bartsch C., Medeiros-Domingo A., Berger W., Haas C. (2018). Exome analysis in 34 sudden unexplained death (SUD) victims mainly identified variants in channelopathy-associated genes. Int. J. Legal. Med..

[13] Qui H., Li W.H., Zhang H.S., Zhou G.X., Li P.W. (2021). Torsades de pointes episode in a woman with high-grade fever and inflammatory activation: A case report. World. J. Clin. Cases..

[14] Tan H.V., Duff H., Gerull B., Sumner G. (2015). Early repolarization syndrome: A case report focusing on dynamic electrocardiographic changes before ventricular arrhythmias and genetic analysis. Heart. Rhythm. Case. Rep..

[15] Forleo C., D’Erchia M.A., Sorrentino S., Manzari C., Chiara M., Iacoviello M., Guaricci I.A., Santis D.D., Musci L.R., La Spada A. (2017). Targeted Next-Generation Sequencing detects novel gene-phenotype associations and expands the mutational spectrum in cardiomyopathies. PloS One.

[16] Johnson N.J., Ackerman J.M. (2009). QTc: How long is too long?. Br. J. Sport. Med..

